# Supplementary material for: Sphingosine Kinase 1 Deficiency in Smooth Muscle Cells Protects against Hypoxia-Mediated Pulmonary Hypertension via YAP1 Signaling
Source: Int J Mol Sci. 2022 Nov 22;23(23):14516. doi: 10.3390/ijms232314516 (PMC9736859; doi:10.3390/ijms232314516)
Supplement: Supplementary file 1 [file ijms-23-14516-s001.zip › ijms-1974956-supplementary.pdf]

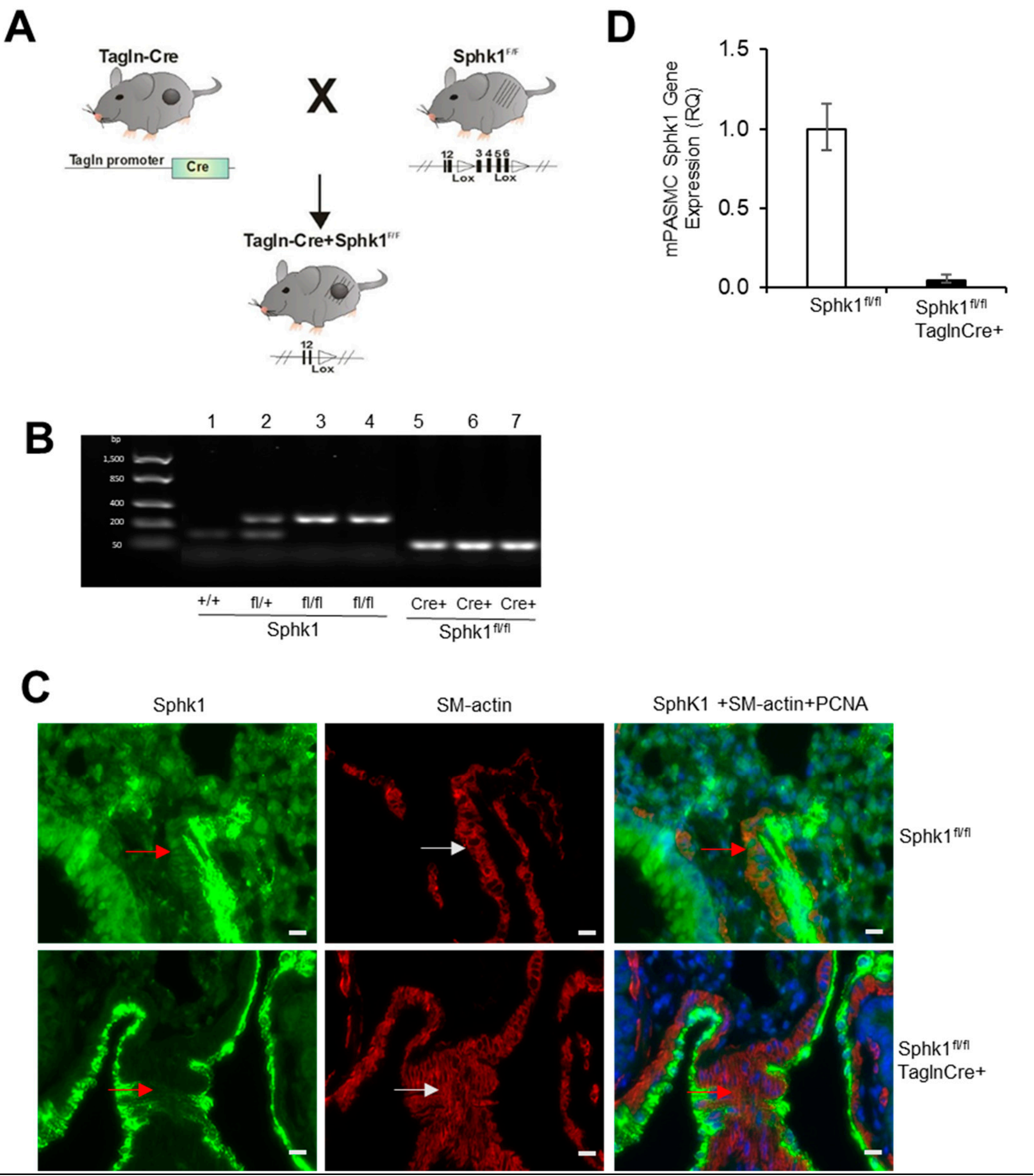

Supplemental Figure S1. Smooth Muscle Specific Deletion of Sphk1 in Sphk1$^{fl/fl}$ Tagln Cre+ mice. A. Sphk1$^{fl/f}$ mice were crossed with homozygous Tagln Cre+ mice resulting in the first familial generation (F_1) having one Sphk1 floxed allele and one Tagln Cre+ allele. F_1 x F_1 generated Sphk1$^{fl/fl}$ Tagln Cre+ mice in the F_2 generation. The *Sphk*1 gene has loxP sites flanking exon 3-5. The Tagln promoter induces smooth muscle specific expression of Cre recombinase resulting in conditional knockout of Sphk1 in smooth muscle cells. B. PCR based genotyping showing the expected DNA products for wild-type and targeted alleles. The following lanes are DNA from the same mouse: (2,5), (3,6) and (4,7). C. Immunofluorescent images of lung tissue from Sphk1$^{fl/fl}$ and Sphk1$^{fl/fl}$ Tagln Cre+ mice showing an absence of Sphk1 expression in the smooth muscle layer of Sphk1$^{fl/fl}$ Tagln Cre+ mice. Red arrows denote Sphk1 and white arrows denote smooth muscle layer. D. Real-time PCR showing that Sphk1 mRNA expression is decreased in PASMCs isolated from Sphk1$^{fl/fl}$ Tagln Cre+ compared to Sphk1$^{fl/fl}$ mice.
